# Radiomics of pituitary adenoma using computer vision: a review

**DOI:** 10.1007/s11517-024-03163-3

**Published:** 2024-07-16

**Authors:** Tomas Zilka, Wanda Benesova

**Affiliations:** 1 Saint Michal’s Hospital, Bratislava, Slovakia; 2 Slovak University of Technology in Bratislava, Bratislava, Slovakia; 3 Masaryk University, Brno, Czech Republic

**Keywords:** Radiomics, Pituitary adenoma, Computer vision, Artificial intelligence, Machine learning, Deep neural networks

## Abstract

**Abstract:**

Pituitary adenomas (PA) represent the most common type of sellar neoplasm. Extracting relevant information from radiological images is essential for decision support in addressing various objectives related to PA. Given the critical need for an accurate assessment of the natural progression of PA, computer vision (CV) and artificial intelligence (AI) play a pivotal role in automatically extracting features from radiological images. The field of “Radiomics” involves the extraction of high-dimensional features, often referred to as “Radiomic features,” from digital radiological images. This survey offers an analysis of the current state of research in PA radiomics. Our work comprises a systematic review of 34 publications focused on PA radiomics and other automated information mining pertaining to PA through the analysis of radiological data using computer vision methods. We begin with a theoretical exploration essential for understanding the theoretical background of radionmics, encompassing traditional approaches from computer vision and machine learning, as well as the latest methodologies in deep radiomics utilizing deep learning (DL). Thirty-four research works under examination are comprehensively compared and evaluated. The overall results achieved in the analyzed papers are high, e.g., the best accuracy is up to 96% and the best achieved AUC is up to 0.99, which establishes optimism for the successful use of radiomic features. Methods based on deep learning seem to be the most promising for the future. In relation to this perspective DL methods, several challenges are remarkable: It is important to create high-quality and sufficiently extensive datasets necessary for training deep neural networks. Interpretability of deep radiomics is also a big open challenge. It is necessary to develop and verify methods that will explain to us how deep radiomic features reflect various physics-explainable aspects.

**Graphical abstract:**

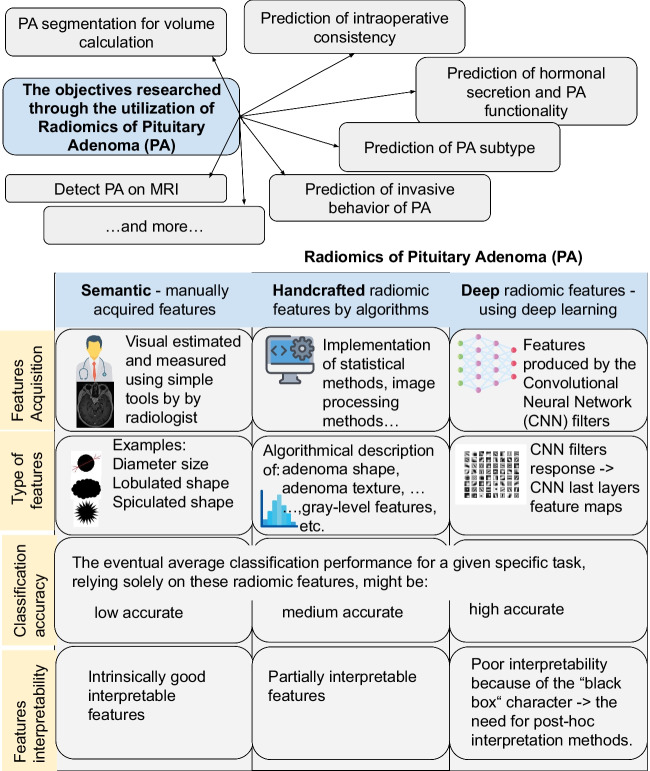

##  Introduction

**Sellar tumors** Sellar tumors are approximately 10–15% among all intracranial neoplasms. The term “sellar” is derived from their anatomical localization—they are located in the area of sella turcica on the base of the skull. Sellar region is a complex topographic-anatomical area, with an abundance of important structures such as optic chiasm, carotid arteries, cavernous sinus, and pituitary gland.

**Pituitary adenoma (PA)** The most common sellar neoplasm is the PA. This benign tumor originates in the pituitary gland. In clinical practice, the most relevant classification of PA is functioning (FPA) and non-functioning (NFPA) according to the absence or presence of oversecretion of pituitary hormones. In most cases, the presenting symptom of NFPA is visual loss (usually worsening of peripheral vision), but the symptomatology only occurs when the size of a PA is large enough to compress the optic chiasm or nerves. Patients with smaller PAs with no compression of optic apparatus may remain asymptomatic for a long time. Only a minority of patients with NFPA have significant hypopituitarism as a dominant presenting symptom. Therefore, NFPAs usually require treatment only when large or growing on serial imaging. On the other hand, FPAs require treatment irrespective to their size because their hormonal overproduction (which can cause a variety of serious symptoms) is not size-correlated. Hence, even a few millimeters small FPA may be indicated for treatment.

PA is a multidisciplinary disease, and its proper management requires the cooperation of endocrinologists, neurosurgeons, ear, nose, and throat (ENT) doctors, etc. When indicated for treatment, the majority of PA require surgical resection (usually endoscopic endonasal). Prolactinomas, a sub-type of FPA-secreting prolactin, represent an exception by being first treated pharmacologically with dopamine agonists. Management of PAs is not a single-session process, but rather a complex algorithm requiring long-term follow-up. Especially challenging are cases with recurrence or growing postoperative residuum. Surgically inaccessible residuals may be treated with radiotherapy. Slow growth of small residuals may be controlled pharmacologically in specific cases.

The dynamic character of PA requires a particularly accurate assessment of the natural course of this disease. Therefore, the role of computer vision and AI is very promising.

**Computer vision (CV) in the radiology**

Computer vision is a scientific field focused on the automatic extraction of information from image data. Hence, it can also prove to be highly valuable in obtaining various types of information from radiological images such as MRI or CT scans. For instance, in the case of PA, methods of computer vision can precisely delineate tumor boundaries through segmentation, assess and predict disease progression over time, gather additional biomarkers like radiomic features (see chapter 2.1), and more.

Computer vision is typically based on applied methods of machine learning (ML), where ML algorithms give computers the ability to learn without being explicitly programmed for the concrete solved task but that allow computers to solve the task by learning from the data.

So-called “traditional ML approaches” typically operate based on the concept of acquiring hand-crafted features, which are subsequently employed as input data for the decision-making unit: the classifier. These traditional ML approaches were highly preferred in the era before the arrival of deep neural networks (DNN), which brought significant and highly positive progress in the ML and application of computer vision techniques over the past decade. DNN highly overcome the precision and robustness of the traditional approach of ML.

Today’s modern methods of DNN are able to handle the robust task of segmentation as well as obtaining radiomic features with high accuracy, but a very important prerequisite is to have a well-prepared annotated and sufficiently large dataset of suitable images for training a deep neural network. There are already numerous relevant publications presenting the ability of AI to aid in the management process of PA by improving the prediction of the clinical behavior of these lesions.

In this paper, we analyzed publications that researched the application of computer vision methods and radiomic features calculation to obtain information about a PA from radiological images.

**Our contribution**

- We analyze the current state of the art of the research related to radiomic features of PA.
- We provide a systematic review of publications related to research with PA radiomics or other automated information mining related to PA from radiology data using methods of computer vision.

## Radiomics

### Radiomics definition

The term “Radiomics” describes the extraction of high-dimensional features (feature vectors) also called “Radiomic features” from digital medical images [8]. The prefix “radio-” refers to the use of radiological images, which means mainly data of computed tomography (CT), positron emission tomography (PET), and magnetic resonance imaging (MRI), as input data. Hence, radiomics performs a quantitative characterization of radiological images to identify image biomarkers.

The goal of the mining of these features is to provide relevant information suitable for decision support which can cover various objectives. These are mainly decision tasks; in the case of PA, it could be for instance: the determination of PA functionality, prediction of hormonal secretion, prediction of treatment response, etc.

**Fig. 1:**
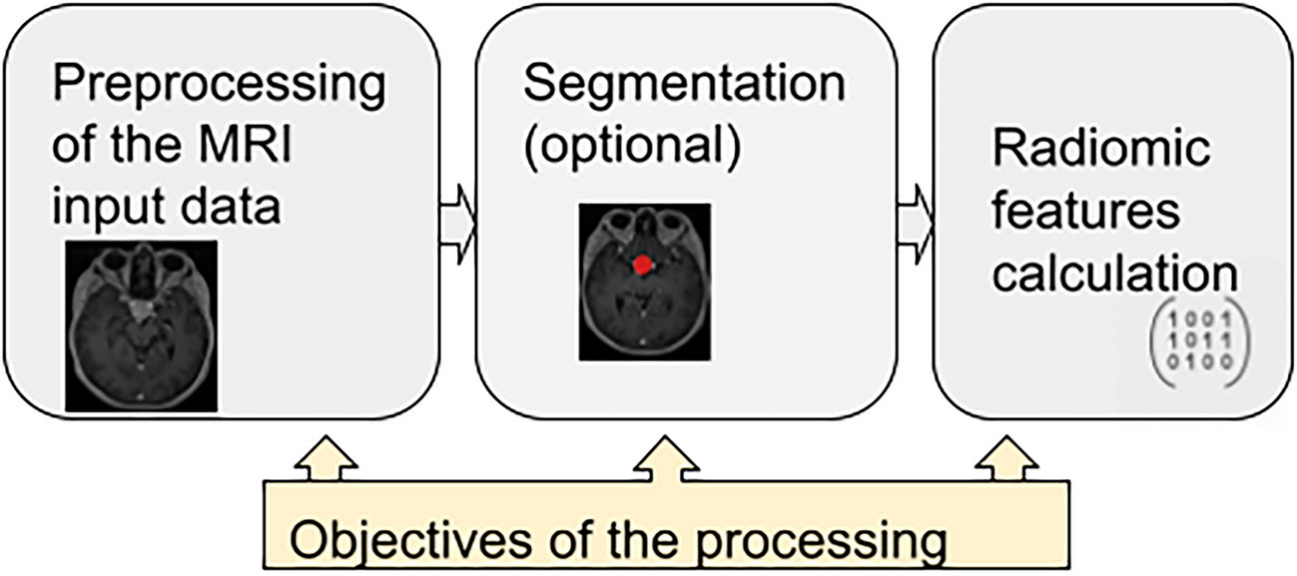
Generic pipeline of radiomics calculation

**Fig. 2:**
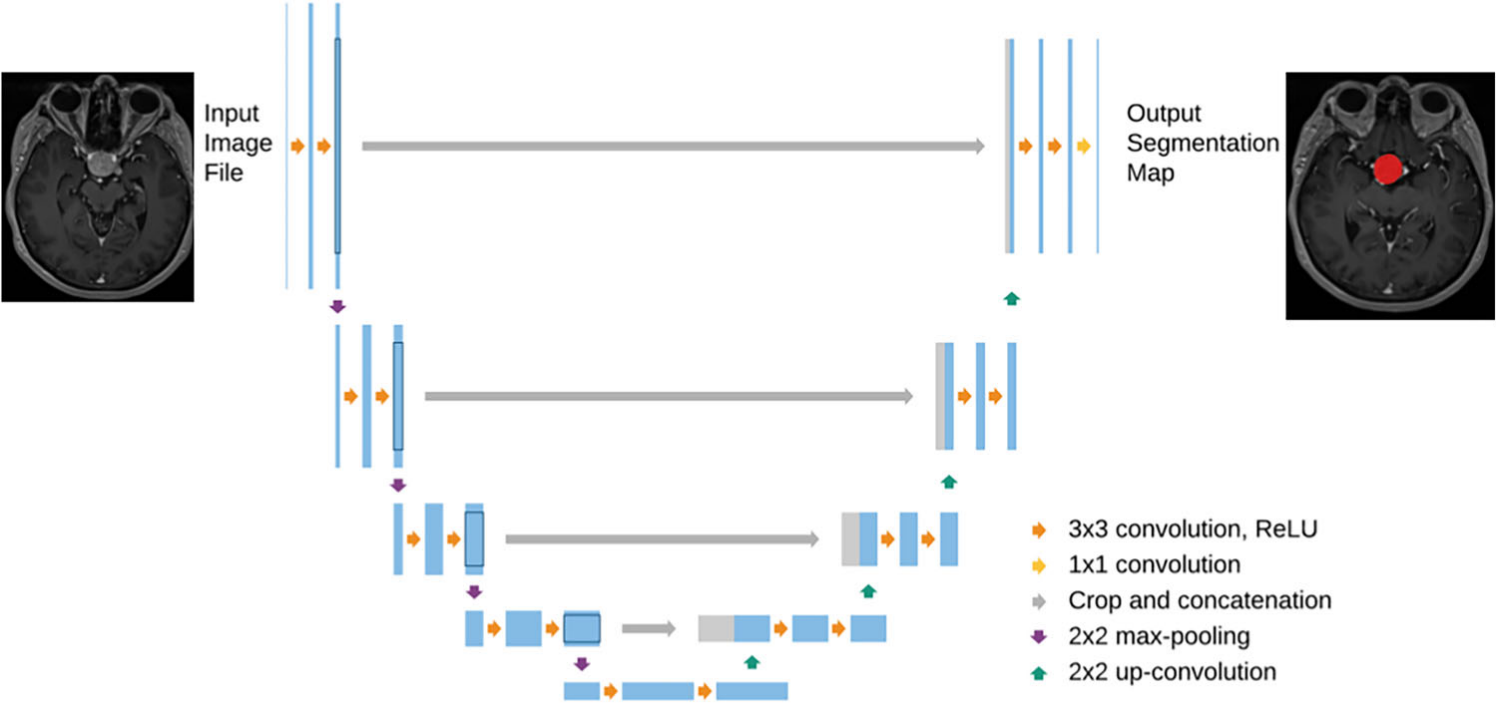
Basic U-net architecture [26]

## Generic pipeline of radiomics calculation (Fig. 1)

### Preprocessing of the MRI input data

MRI images can be pre-processed for the purpose of noise removal, brightness correction of intensity, data normalization, etc. An important step can also be cropping the 3D image to the relevant part (region of interest—ROI), which in the case of PA is relatively easily processed because of the anatomical position of the PA.

### Segmentation

Adenoma segmentation is generally an optional part of the process, but if we want to calculate the features describing the shape of the adenoma, accurate segmentation is essential.

**Segmentation using traditional computer vision approach** Segmentation can be carried out manually by drawing the contours of the adenoma by a human expert (this is very expensive and time-consuming), or they can be semi-automatic, such as using the thresholding method (however, the accuracy of this approach is insufficient for many use-cases) or also may be used more advanced computer vision approaches as for instance “graph cut” method.

**Segmentation using deep learning (DL) approach** Also in the case of segmentation, the development of deep neural networks has brought significant progress. Very popular and well-working is the U-net neural network architecture and its modifications for the segmentation tasks. Much research as well as practical experiences confirm the quality and reliability of segmentation using the U-net architecture [30] (Fig. 2).

A sufficiently large annotated dataset for training the U-net neural network is crucial for a successfully trained model.

For some tasks, e.g., general brain tumor segmentation, extensive datasets such as BRATS [19] were created and are publicly available for research purposes. Hence, several well-trained segmentation models on this dataset are already available. However, when training a DNN automatic segmentation model applied to a new medical task, such as the PA segmentation task, we have to deal with the creation of a new annotated dataset.

The problem of dataset generation and the small amount of annotated data available for training is a remaining challenge. To mitigate this problem, some strategies are helpful as for instance “transfer learning” (where the network is pre-trained on different data) and data augmentation methods to generate more data for training.

## Radiomic features and their calculation

As mentioned in the chapter 2.1, radiomics is concerned with the extraction of quantitative metrics, the so-called radiomic features, within radiological images. Radiomic features capture tissue and lesion characteristics and may, alone or in combination with demographic, histologic, genomic, or proteomic data, be used for clinical problem-solving [17].

**Fig. 3:**
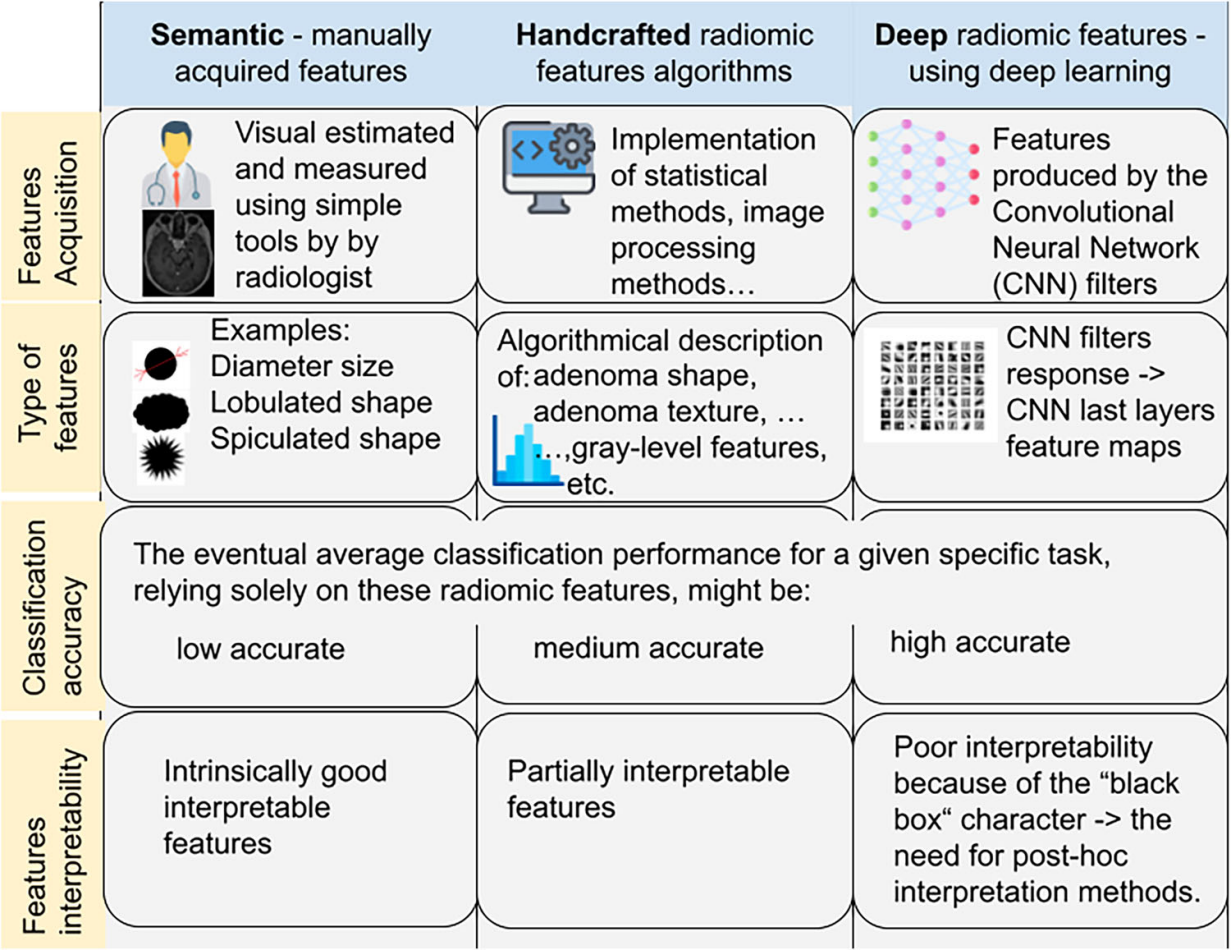
A comparison of semantic, handcrafted radiomic, and deep radiomic features [20, 39]

Radiomic features can be obtained by different approaches. Figure 3 illustrates approaches of radiomics mining: from simple estimation by a radiologist (left), over the traditional approach of machine learning using handcrafted features (middle) to deep radiomic features using DL (right).

**Fig. 4:**
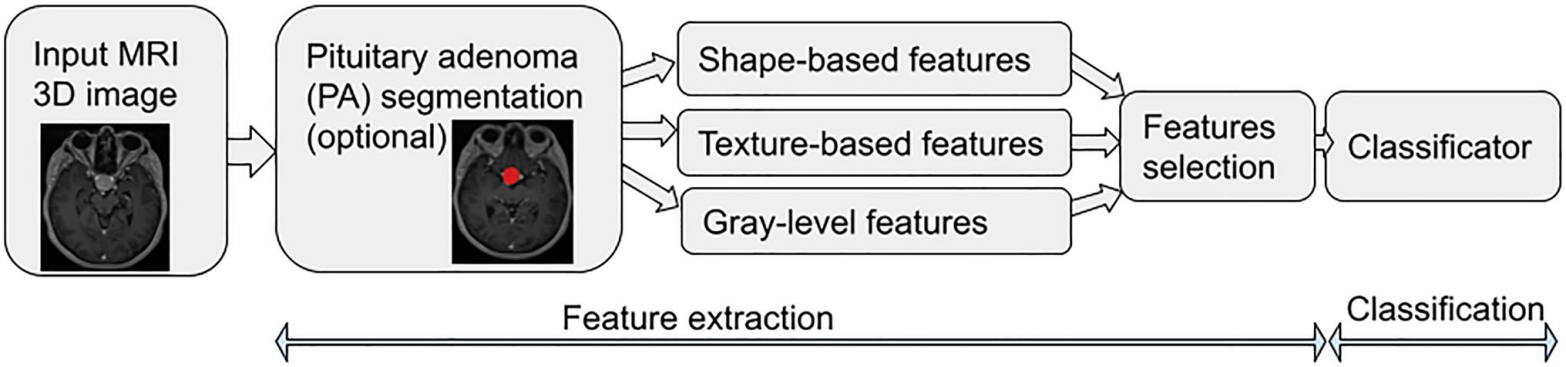
Pipeline of handcrafted features calculation

### Semantic manually acquired features (Fig. 3 left)

This means the acquisition of radiomic features manually by radiologists using simple support tools such as distance measurement in a radiological image, etc. In this way, only very limited semantic features like size and visual shape description (lobulation, spiculation, etc.) can be manually measured or visually evaluated by human experts.

#### Interpretability of manually acquired semantic features

The advantage of this limited approach is the very good interpretability of these radiomic features since each feature itself is designed to describe one radiomic property that it represents. This leads to the fact that the features are intrinsically interpretable.

### “Handcrafted algorithms for the calculation of radiomic features (Fig. 3 middle)

Handcrafted radiomic features calculation (Fig. 4) uses a traditional computer vision approach, i.e., methods prominent, especially in the era before deep learning.

#### Features extraction

“Handcrafted” in this case means that when designing the calculation algorithm, the analyst focuses on certain intuitively relevant features, such as algorithmic description of the shape of the adenoma, statistical description of the texture of the adenoma, mathematical description of brightness changes in the area of the adenoma, etc. For the calculation of these features, he proposes “by-hand” an algorithm for their calculation using the traditional image processing and computer vision methods.

**PyRadiomics library** A popular implementation tool of handcrafted radiomic feature calculation is the library PyRadiomics radiomic and deep radiomic features [34], which provides calculation of shape and texture-based radiomic features in Python. This library includes the implementation following features of an image region of interest (ROI), in this case, a segmented area of the PA:

- Shape-based (2D and 3D) description such as mesh volume, voxel volume, surface area, sphericity, compactness, spherical disproportion, maximum 3D diameter, and more.
- Texture description using statistics features such as energy, entropy, range, percentile, mean absolute deviation, statistics moments, and more.
- Gray-level features such as dependence matrix, zone matrix, and more.

Most features defined in this library are in compliance with feature definitions as described by the Imaging Biomarker Standardization Initiative (IBSI) [47] image. The aim of the authors was to find and standardize the set of characteristics present in medical imaging. They proposed 169 standardized radiomic features, which have been evaluated as good to excellent regarding reproducibility for radiomic features using MRI, fluorine 18 fluorodeoxyglucose PET, and CT images obtained in 51 patients with soft-tissue sarcoma.

#### Features selection

As mentioned, these radiomic features need to be assessed from the point of view of contributing relevant information for the given objective. Since it is usually difficult to intuitively predict which combinations of features are most relevant for a given task, the common strategy used is to calculate a large number of various features, which are then selected in the next step. This is a common engineering approach to calculate an excess number of features and then, in the next step, based on their mutual correlation, select a subset of features that are the most relevant ones.

Therefore, a feature selection method could be applied, where only a subset of all counted features is selected. However, this feature selection step is optional; it is also possible to put all the counted features to the classifier which determines and manages their relevance.

#### Interpretability of the handcrafted radiomic features

Our goal should be not only the calculation of the relevant radiomic features but also the interpretation of their physical nature, which is an important task because of understanding on which characteristics are the selected relevant radiomic features focused, e.g., on a certain description of the shape of the adenoma or on a certain mathematic description of the texture of the adenoma.

In the case of the handcrafted features, it is a relatively good possibility to interpret the radiomic features according to the algorithm of their calculation. This is relatively well understandable and transparent if the number of the selected features is small. In this case, we can derive the contribution of individual radiomic features to their overall importance and thereby intuitively understand and interpret their meaning in relation to explainable parameters.

#### Classification

Classification is an essential task of supervised machine learning which is used to classify an unknown sample of data into a known group of the data: the so-called class. In most cases, the previously calculated features are used as input data of the classifier; hence, the classification part follows the features extraction (and optional features selection) part in a pipeline of the traditional supervised machine learning classification approach (see Fig. 4). Many classification algorithms have been published, and among the most important are following classifiers:

*Naive Bayes (NB)* classifier is a probabilistic-based classification algorithm based on Bayes’ Theorem. *Support Vector Machine (SVM)* algorithm works by creating a hyper-plane in an n-dimensional feature space that separates the data points belonging to different classes. *Linear and Quadratic Discriminant Analysis (LDA/QDA)* are two classic classifiers, with a linear and a quadratic decision surface in the feature space, respectively. *k-Nearest Neighbors(kNN)* is also a supervised learning classifier that uses the proximity of the features in the feature space to classify the data sample. To solve a classification problem, the kNN algorithm assigns a class label based on a majority vote of k-nearest neighbors in the feature space. *Decision tree (DT)* is a well-interpretable classification that builds a flowchart-like tree hierarchical structure. Each internal node denotes an *if-then-else* decision. *Random Forest (RF)* utilizes ensemble learning, which is a technique that combines several classifiers in one classification task, typically an ensemble of decision trees obtained by the random selection of a group of variables from the variable space. *Gradient Boosting Machines (GBM)* apply powerful methods that can effectively capture complex non-linear function dependencies. Finally, *multi layer perceptron (MLP)* is a relatively shallow feed-forward neural network with a limited number of fully connected hidden layers.

**Fig. 5:**
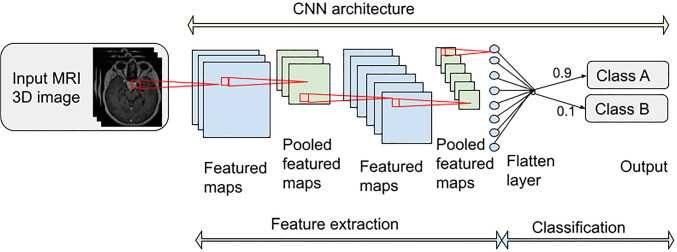
A typical convolutional neural network (CNN)[39]

The choice of a suitable classifier for a given task should be verified experimentally, while prior experience in designing decision-making processes can be valuable.

### Deep radiomic features (Fig. 3 right)

The term “deep” in this case refers to the use of DNN, i.e., neural networks that have a deep architecture, which means that many layers of neurons are arranged one behind the other.

*Convolutional neural network (CNN)* (Fig. 5) represents a specialized type of deep neural network tailored for image processing. Consequently, CNNs are well-suited for the automated generation of radiomic features using deep neural networks, since they contain the so-called convolution filters.

The parameters of these convolution filters are automatically adjusted during the process of training the neural network using the training data. This automatic setting of filter parameters crucial differs from the hand-crafted features creation described previously. Hand-crafted can also contain various filters, but in contrast to the CNN, these filter parameters have to be set manually.

In the process of classifying an input image, the feature maps and activation maps sequentially represent the deep radiomic features as follows: from low-level features in the initial convolution’s layers, progressing through medium-level features in the intermediate convolution’s layers, to high-level features in the final convolution’s layers.

Then, these features can be used as radiomic features, where high-level features from the last convolution layers are especially suitable.

#### Interpretability of deep radiomic features

As mentioned in chapter 4.2.3, an important factor is not only the calculation of suitable radiomic features but also the possibility of interpreting their physical meaning.

Since deep neural networks with billions of parameters behave like “black boxes” and are inherently “non-transparent,” deep radiomic features are challenging to explain. To make them explainable, we need to subsequently apply various interpretability methods. Research in the field of deep neural network interpretability is extensive and has already yielded many suitable approaches.

Well-known approaches are visualization methods, which reveal the part of the input image that was most relevant for network decision-making. These are the so-called “saliency maps.”

Another post-hoc explanation approach is the method known as Concept Activation Vectors (TCAV). TCAV can answer questions related to the given concept, for example, the question of how a specific type of tumor shape is significant for the DNN decision process? [10].

We can conclude that research in the field of interpretability of DNN is a big open challenge and is the focus of various research teams.

## The survey: methods and materials

To present the current state of the research focused on the problem related to radiomics of PA using computer vision, we analyzed 34 published scientific works. These researched publications are listed in tabular form in Table 1. Our primary focus has been on comparing the publications from the perspective of the size of the utilized dataset, the type of task being addressed, the usage of segmentation, the utilized features, and their quantity, the type of classifier, and the evaluation of the results.

**Table 1:** Overview of the analyzed papers sorted by year of the publications

Ref.	Title	Year	Size of dataset	Task	Segment.	Features	Classif.	Results
[3]	*Pituitary adenoma segmentation*	2010	10	Segmentation only				
[46]	*Preoperative volume determination of PA*	2011	10	Volume determination	Semi-auto	1 Volume	–	DICE 0.76
[4]	*Adenoma volumetry with 3D slicer*	2012	10	Volume determination	Semi-auto	1 Volume	–	DICE 0.82
[42]	*Non-invasive radiomics approach potentially predicts...*	2018	112	Predicts non-functioning PA	Manual	741	SVM	AUC 0.83
[32]	*Utility of deep neural networks in predicting gross-total resection...*	2018	140	Predict GTR	Manual	7	DNN	AUC 0.96
[43]	*Non-invasive radiomics approach potentially predicts non-functioning PA subtypes before surgery*	2018	112	Predict NCA who are more likely to respond to neo-adjuvant RT	Manual	741	SVM	AUC 0.83/0.80 (valid./test.)
[6]	*Development and validation of an MRI-based radiomic signature...*	2019	108 + 55	Prediction of treatment response	Manual	7 from 1395 gabor features	SVM	AUC 0.808
[21]	*Preoperative prediction of cavernous sinus invasion...*	2019	97+97	Prediction of CS invasion	Manual	3 from 2553 Sphericity	SVM	AUC 0.826
[5]	*Preoperative noninvasive radiomics approach predicts tumor consistency...*	2019	100 + 58	Predict PA consistency	Manual	4 from 4683	RF	AUC 0.83
[33]	*Prediction of high proliferative index in pituitary macroadenomas using MRI...*	2019	89	Predict Ki-67% (high/low)	Manual (ITK Snap)	1128	kNN	Accur.91.6%
[11]	*Predicting response to somatostatin analogues in acromegaly: ML-based...*	2019	47	Predict response FA to somatostatin analogues	Manual	828	qTA, k-NN, DT	AUC 0.847
[40]	*Preoperative evaluation of tumor consistency in pituitary macroadenomas...*	2019	55	Predict consistency	Manual	6 histogram f. (PyRadiomics)	MLP	AUC 0.71
[9]	*Prediction of response to stereotactic radiotherapy for NF PA...*	2020	62+31	Predict response to Stereot. RT	Manual	4 from 1208 area, wavelet f	SVM	AUC 0.89
[2]	*Prediction of pituitary adenoma surgical consistency...*	2020	89	Predict intraoperative consistency	Manual	14 from 1118	DT	Accur. 93%, AUC 0.99%
[24]	*A machine learning model to precisely immunohistochemically classify PA...*	2020	235	Predict immunohistochemical parameters of PA	Manual	788	SVM, kNN, NBs	AUC 0.93
[16]	*MRI radiomics for the prediction of recurrence in patients with clinically NFPA...*	2020	27	Predict recurrence	Semi-auto (3D slicer)	10 from 255	kNN, RF, LR, SVM, MLP	AUC 0.962
[25]	*A novel diagnostic method for PA based on MRI using a CNN...*	2020	149	Detect presence of PA	Manual bounding box	Deep radiomics (CNN)	CNN	Accur.91–97%
[22]	*Radiomics model predicts granulation pattern in growth hormone-secreting pituitary adenomas*	2020	69	Predict granulation pattern	Semi-auto (3D slicer)	4 from 214 Shape+Statist. fea	LR	Accur.73.7% AUC 0.834
[44]	*Radiomics approach for prediction of recurrence in non-functioning...*	2020	50	Predict progression / recurrence in NFPAs	Auto + man. correction	32 statist. + 75 texture f	SVM	Accur. 82% AUC 0.78
[29]	*Three-dimensional semantic segmentation of PA based on the DL...*	2021	317	Segmentation only	Auto (DNN: U-NET)	–	–	DICE 80%
[27]	*Radiomics analysis allows for precise prediction of silent corticotroph adenoma.*	2021	242 + 60	Predict SCA	Manual	631	SVM, LDA, RF, MLP	AUC 0.92
[13]	*Image-driven classification of functioning and nonfunctioning PA by deep CNN*	2021	100+85	determine PA functionality FPA / NFPA	Auto (DNN)	Deep radiomics (CNN)	CNN	DICE 81% AUC 0.80
[23]	*Radiomics with ensemble ML predicts dopamine agonist response in patients...*	2021	141+36	Predict response to DA (responder/nonresponder)	Semi-auto (3D slicer)	107 (PyRadiomics)	RF, DT, GBM, QDA/LDA	AUC 0.81
[15]	*Development and validation of a deep learning algorithm...*	2021	780 + 195 + 545	Detect microadenoma on MRI	Auto Bounding box (DNN)	Deep radiomics (CNN)	CNN	Accur.94.3% AUC 0.981
[38]	*MR-based radiomics for differential diagnosis between cystic PA and Rathke cleft cyst*	2021	172 + 43	Predict Cystic PA/Rathke cleft cyst	Manual	20-40 from 330 (PyRadiomics)	SVM, MLP, AdaBoost, RF	Accur.76.7% AUC 0.848
[1]	*Multivariable diagnostic prediction model to detecting hormone...*	2022	130	Predict hormonal secretion	Manual	851	MLP	AUC 0.95
[7]	*A CNN model for detecting sellar floor destruction of PA on MRI scans)*	2022	695	Detect sellar floor destruction invasive/non invasive	Manual slice select	Deep radiomics (CNN)	CNN	Accur.96% AUC 0.98
[41]	*Prediction of high infiltration levels in pituitary adenoma...*	2022	176 + 20	Predict high infiltration levels	Semi-auto (3D slicer)	19 from 4120	SVM	Accur.85% AUC 0.73
[45]	*A preoperative MRI-based radiomics-clinicopathological...*	2022	74 + 94	Predict recurrence	Manual	4 from 1130	MLP	AUC 0.783
[36]	*Radiomic features on multiparametric MRI for preoperative eval...*	2022	156	Predict consistency	Auto (DNN)	388	SVM, RF	Accur.87% AUC= 0.90
[18]	*Radiomic analysis of preoperative magnetic resonance imaging...*	2023	52	Predict PA consistency	Manual	10 from 694 statist. fea	SVM and RF	AUC 0.956
[37]	*Radiomics model and clinical scale for the preoperative diagnosis...*	2023	260	Predict SCA	Manual	38 (PyRadiomics)	MLP,DT,... (10 ML)	Accur.83.85% AUC 0.931
[14]	*Preoperatively predicting Ki67 expression in pituitary adenomas using deep...*	2024	1214	Predict recurrence, classify HG and LG	Auto (DNN)	18+15+11 nomogram	DT	AUC: 0.831 (Valid.) 0.825 (Test)
[28]	*Is radiomics a useful addition to magnetic resonance imaging...*	2024	149+73	Predict high-risk/low-risk NF PitNETs	Manual	max 8 from 100 (PyRadiomics)	SVM, LR, RF, MLP	Accur. 67% AUC 0.76

These assessed parameters of the compared methods follow the pipeline scheme reflecting a general approach to analyzing pituitary adenoma using AI. The pipeline begins with MRI image input, which is followed by PA segmentation, extraction of radiomic features, and classification (Fig. 1). The analyzed works follow the use of radiomics for various objectives and tasks.

### Input data—datasets

The published experiments primarily utilize T1C or T1C +T2 MRI data as input. When the authors solely provide the dataset’s size, it is presented as a single value, denoted as S1, in Table 1, column “Size of dataset.” In such cases, the authors do not provide explicit details about the division of the dataset into training, validation, and testing subsets. If two values, S1 + S2, are listed in the table, they pertain to the sizes of the training and validation datasets. In this scenario, the validation data seem to have been repurposed for testing purposes. The format S1+S2+S3 encompasses three dataset sizes: number of the training data, number of the validation data, and number of the test data.

The experiments show significant differences in the total size of the datasets employed. Earlier publications employed a limited dataset size (e.g., 10 MRI scans), while more recent works employed notably larger datasets. The dataset’s magnitude is a pivotal parameter of the experiment, offering insights into the statistical significance of the results and the potential susceptibility to over-fitting of the training.

### Tasks and objectives of the studies

The analyzed works, which deal with the utilization of artificial intelligence (AI) and computer vision for pituitary adenomas (PA) analysis, can be categorized based on the objectives they pursued, as follows:

**Only adenoma segmentation for volume calculation** [3, 4, 29, 46] The clinical application of such works is limited. Fast and automatic volume calculation may provide some insight into the future course of the disease (extremely large tumors being more prone to complications during treatment). Possible use is in follow-up and identification of growing PA.

**Detection of a presence or absence of PA on MRI** [15, 25] Both studies used a human-defined bounding box delineating the sellar area and subsequently convolutional neural network (CNN) as a classifier determining the presence or absence of a microadenoma. Authors in [15] achieved 94.3% diagnostic accuracy and 0.981 AUC score, and authors in [25] reported overall accuracy of 91%, sensitivity of 92.2%, and specificity of 75.7%.

Microadenomas, due to their small size, may be missed by radiologist. Automated microadenoma detection can increase the probability of microadenoma discovery.

**Prediction of an invasive behavior of PA** An interesting retrospective study from [21] dealt with cavernous sinus (CS) invasion of PA marked as Knosp grade 2 and 3. They implemented a manual segmentation and 97 patients for training and testing. A support vector machine (SVM) was used as a classifier whether the tumor would or would not invade CS. Predictions were compared to intraoperative findings. AUC 0.852 and 0.826 for the training and test sets were achieved.

Invasive behavior of PA from different perspectives was analyzed by [7]. They focused on the detection of sellar floor destruction by PA. There were no segmentation used, but for every patient, a single MRI slice with sellar floor invasion (SFI) was picked by an experienced neurosurgeon after the patient underwent resection which confirmed the SFI. A deep neural network CNN was implemented and trained to produce binary output (invasive/non-invasive). The testing set presented excellent performance, with a model prediction accuracy of 96%, a sensitivity of 0.964, and a specificity of 0.958.

Prediction of PA invasiveness has a great clinical value. Invasive PA tends to grow into hardly accessible anatomical compartments such as cavernous sinus. Therefore, they carry a higher risk of having post-operative residuum. In the case of functioning PA, postoperative residuum usually means persistence of hormonal hyperproduction, hence failure of treatment. Residuum in non-functioning PA may become growing and lead to relapses of symptoms, mainly visual if they reach optic nerves or chiasm.

**Prediction of a PA subtype (histological, immunological)** [43] focused on identifying null cell adenomas which are a subtype of non-functioning PA that are more likely to respond to neo-adjuvant radiotherapy. The study had 112 patients (training set: *n* = 75; test set: *n* = 37). Manually segmented 2D MRI scans were processed by the SVM classification yielding area under the curve (AUC) values of 0.8314 and 0.8042 for the training and test sets, respectively.

Interesting work from [24] tried immunohistochemically to classify PA’s based on preoperative MRI. They used multiple 2D scans, manually segmented, overall from 255 patients. The study also used a neural network to identify 788 radiomic features. Results were compared to immunohistochemical findings. Results reported AUC 0.93 in clustering patients to Tpit, Pit-1, and SF-1 types.

The authors in [27] implemented radiomic analysis for the detection of silent corticotroph adenomas (SCA), which represent a subtype of NFPA that tend to have a more invasive behavior. Patients with SCA do not have laboratory hypercortisolism; the immunohistochemical confirmation is Tpit positivity. The authors used manual segmentation of MRI scans, and the dataset consisted of 146 patients with SCAs and 156 patients with non-SCAs. A total of 631 relevant radiomic features were selected from 1919 calculated features. The classification was binary—SCA/NonSCA, and the ensemble algorithm presented the largest AUC of 0.927.

**Prediction of hormonal secretion and PA functionality** The ability to predict a consistency of pituitary adenoma has several implications. It provides insight into intraoperative findings. A firm tumor, especially when occupying a hardly accessible area or neighboring crucial anatomical structures requires longer surgery, an experienced team, and possesses higher risk. Also, it is more probable for a firm tumor to have a post-operative residuum than for a soft one.

The prediction of the hormonal subtype has a high clinical value. It is crucial to know if PA produces excess amounts of some pituitary hormones. These adenomas are considered functional and have specific management. The main difference is that even very small functioning pituitary adenomas may be indicated for resection, aiming to normalize hormonal hyperproduction, while small non-functioning PA may remain untreated.

Prediction of hormonal secretion and PA functionality was a goal of [1, 13]. First mentioned studied 130 patients, used manual segmentation, and calculated 851 radiomic features. Multilayer perceptron achieved 95% accuracy in classifying whether a pituitary adenoma is a prolactinoma.

The second study showed broad implementation of neural networks. They designed a model for segmentation which yielded a Dice score of 0.8093 for the testing dataset. Another model had aimed for classification (functioning PA/non-functioning PA) and achieved AUROC 0.8478. There were hand-crafted radiomic features used.

**Prediction of intraoperative consistency** To predict whether the PA will be soft or firm according to MRI scans was tried by [2]. They had 89 patients in total. Their study implemented manual segmentation. Radiomic features were calculated and evaluated resulting in the selection of 14 relevant RF out of 1118. Classification was done by a decision tree (DT) achieving 93% accuracy.

The similar goal had work of [5]; however, they limited their scope to patients with functioning adenomas causing acromegaly. Segmentation was manual, and 4 key radiomic features were selected from 4683 initially calculated. They had 150 patients (100 primary cohort, 58 validation) to produce the radiomics model, which incorporated both the radiomics signature and Knosp grade. Results displayed favorable discriminatory capacity and calibration, and the AUC was 0.83 (95% confidence interval, 0.81-$-$0.85) and 0.81 (95% confidence interval, 0.78-$-$0.83) in the primary and validation cohorts, respectively.

Mendi, Bökebatur Ahmet Raşit et al. [18] analyzed surgical notes of 52 patients and were able to predict high consistency of a PA with respectable results (AUC 0.956). The distinction between firm and soft PA was also a goal of Tao Wan et. al [36]. They proved that the model trained on 108 patients which implemented 388 radiomic features calculated from T1, T2, and T1CE coregistered and automatically segmented MRI scans had the best performance (AUC 0.9).

*Prediction of a recurrence* represents a very important task in the perspective of pituitary adenoma patient management itself. Immunohistochemically, the Ki67 percentage has shown to be a reliable predictor of recurrence. In patients with high Ki67 levels (3% and higher), there is a higher chance for a tumor residue to regrow. Hongxia Li et al. [14] tried to predict high versus low Ki67 expression. They implemented an automated segmentation on 1214 cases, used PyRadiomics to generate up to 18 features, and classified utilizing a decision tree (DT) model. The reported DSC was 0.723-$-$0.930.

The paper from Yang Zhang [44] used traditional AI methods on a small sample of 28 patients. MRI scans underwent semi-automated segmentation, selected were simple features, and SVM served as a classifier. In their results, visual disturbance, hypopituitarism, extrasellar extension, compression of the third ventricle, large tumor height and volume, failed optic chiasmatic decompression, and high SVM score was more frequently encountered in patients with recurrent PAs. This paper may be regarded more as a confirmation of well-known risk factors for PA recurrence using AI methods. Interesting research was done by Yu Zhang et al. [45] when they tried to predict PA recurrence over a 5-year horizon. They managed to prove (AUC 0.783) that the most accurate model was a combination of clinical parameters and graphic data—MRI processed by manual segmentation, selection of 4 radiomic features (PyRadiomics), and evaluation by classical methods such as MLP.

The study [16] evaluates the prognostic value of MRI radiomics combined with machine learning to assess recurrence after first surgery in NFPA patients. A retrospective study of 27 patients (10 with recurrence, 17 without) used preoperative 3D T1 contrast-enhanced MR images to extract 255 radiomics features. Radiomics features, gender, age, and remnant tumor tissue were also examined to train five machine learning algorithms that were used to classify recurrence. Accuracy reached up to 96.3%.

**Prediction of gross total resection (GTR)** Achieving GTR means removal of all visible tumor tissue—as assessed either intraoperatively (e.g., via endoscope or operative microscope) or more strictly speaking—with no residual tumor tissue on follow-up MRI scans. GTR correlates with lower recurrence rate. To achieve remission as a favorable result in treatement of functioning pituitary adenomas, it is necessary to remove all hormone-producing adenoma tissue. Therefore, the logic is almost similar to achieving GTR. Paper from [6] analyzes 108 primary and 55 validation cases with emphasis on postoperative hormone levels. Seven radiomic features were selected, and SVM is used as a classifier. Reported AUC was 0.834 and 0.808 on the primary and validation cohorts, respectively, for prediction hormonal postoperative remission.

Staartjes et al. [32] evaluated MRI (pre- and post-operative) from 140 patients. Manual segmentation and assessment of GTW were performed. The deep learning model achieved excellent area under the curve (AUC; 0.96). The authors made an interesting comparison of the model to Knosp classification (higher Knosp grade means higher invasiveness hence lower chance to GTR). An improvement in comparison with the Knosp classification (AUC: 0.87) was noted.

**Differentiation of cystic sellar masses (PA vs Rathke cleft cyst)** Tumors of the sellar region can also be almost completely cystic. In such a case, it is difficult to differentiate between cystic pituitary adenoma (CPA) and Rathke cleft cyst (RCC). The MRI image of both pathologies is very similar. However, these are different diagnoses requiring a similar but not completely identical clinical approach.

Wang et al. [38] designed an interesting study examining 215 patients (105 CPA and 110 RCC) to resolve the aforementioned task. Their ANN classifier was able to discriminate cystic pituitary adenoma from Rathe cleft cyst with an AUC of 0.848.

**Prediction of a response of PA to pharmacotherapy** Only specific types of PA are amenable to pharmacotherapy. One example is growth hormone (GH) secreting pituitary adenomas. Besides surgical removal as the mainstay of treatment, somatostatin analogues (SSA) can be used to reduce tumor volume preoperatively, control hormonal levels pre- and postoperatively. [11] et al. focused on dimension reduction of PA as a response of administration of SSA. They had 24 responsive and 23 resistant patients. Manual slice-by-slice segmentation was utilized. A total of 535 features were selected. k-NN correctly classified 85.1% of the macroadenomas regarding response to SAs with an area under the receiver operating characteristic curve (AUC-ROC) of 0.847.

Prolactinomas represent a specific group within PA, where pharmacotherapy with dopamine agonists (DA) is a first-line treatement. [23] evaluated 177 prolactinoma cases treated with DA and used ML to predict clinical response. Semi-automatic segmentation was performed. A total of 107 radiomic features were calculated using PyRadiomics. Soft voting ensemble classifier has shown superiority over single models in both predictive power and generalization capability (AUC 0.81)

### Segmentation

Segmenting adenomas opens up the opportunity to compute radiomic features associated with the adenoma’s shape. From this point of view, the quality of segmentation appears to be an important factor, especially for hand-crafted features. When dealing with deep features, it is conceivable that the DNN might extract shape-related features pertaining to the adenoma even in the absence of prior segmentation. However, confirming this would require validation through interpretability methods.

Segmentation of PA from MRI data using traditional segmentation methods carries the risk that, if we use an automated procedure, unaccepted segmentation errors will occur. This is probably why a large part of the authors of the analyzed papers were satisfied with manual or semi-automatic segmentation. Semi-automatic segmentation provides some support of manual segmentation process but still requires the manual interaction of a human expert. The vast majority of experiments even used only manual segmentation.

Exceptions are the publications [13, 15, 29], which used a modern segmentation approach using deep learning U-net architecture or DNN object detector to find the bounding box.

This is overviewed in Table 1 in the column named “Segment.”

### Features

In the analyzed experiments, we encounter hand-crafted features in most cases. For the calculation of hand-crafted features, the authors usually have used the PyRadiomics library introduced in the chapter 4.2.1. In most cases, the number of calculated features before feature selection was high (several hundred and more). For the feature selection, the authors have used very different mathematical methods for feature selection, while generally pursuing different goals. For example, Recursive Feature Elimination (RFE) for feature selection [9]. Only recent studies [7, 13, 15, 25] used the calculation of deep radiomic features, i.e., deep learning using CNN.

Features description is summarized in Table 1 in the column named “Features” as follows: In the case of traditional hand-crafted features, the table provides the number of the used features X as follows: If it is only one number in the table, that means the number of the used features is “X.” If there are two numbers in the form “X from Y,” that means Y is the number of all features and X is the number of selected features.

In the case of deep radiomics, the number of radiomic features has not been determined.

**Table 2:** Overview of the best results by solved tasks in the analyzed papers

Tasks and objectives	All Ref.	Best results				
		Year	Ref.	Results	Number of the MRI data	Classif.
Volume calculation only	[3, 4, 29, 46]	2021	[29]	DICE 0.8	317	DNN U-NET
Detect the presence or absence of PA on MRI	[15, 25]	2021	[15]	Accuracy 94.3%, AUC 0.981	780 train + 195val. + 545 test	Deep radiomics (CNN)
Predict invasive behavior of PA	[7, 21, 41]	2022	[7]	Accuracy 96%, AUC 0.98	695	Deep radiomics (CNN)
Predict PA subtype (histological, immunological)	[22, 24, 27, 28, 37, 43]	2020	[24]	AUC 0.93	788	SVM, kNN, NBs
Prediction of hormonal secretion and PA functionality	[1, 13, 42]	2022	[1]	AUC 0.95	545	MLP
Prediction of intraoperative consistency	[2, 5, 18, 36, 40]	2020	[2]	Accur. 93%, AUC 0.99%	89	DT
Prediction of a recurrence	[9, 14, 16, 33, 44, 45]	2020	[16]	AUC 0.962	27	kNN,RF, LR,SVM, MLP
Prediction of the gross-total resection (GTR)	[6, 32]	2018	[32]	AUC 0.96	140	Deep radiomics (CNN)
Predict response to pharmacotherapy	[11, 23]	2019	[11]	AUC 0.847	47	qTA, k-NN, DT
Predict cystic PA/ Rathke cleft cyst	[38]	2021	[38]	Accur.76.7% AUC 0.848	172 train, + 43 test	SVM,MLP, AdaBoost, RF

### Classification

For the classification of handcrafted features, the authors used selected well-known traditional machine learning classifiers (see Sect. 4.2.4). The most represented classificator is popular SVM. Note that the authors of the presented studies [18, 23, 27, 36–38] and [28] applied a combination of more classifiers as an ensemble of classifiers.

**Overview of the used classifiers (traditional approach)**:

- AdaBoost in papers [37, 38], k-Nearest Neighbors (kNN) in papers [16, 24, 33, 37],
- Decision tree (DT) in papers: [2, 11, 14, 23, 37],
- Random forest (RF) in papers [5, 9, 16, 18, 23, 27, 28, 36, 38],
- Logistic regression (LR) in papers [16, 22, 28, 28],
- Multilayer perceptron (MLP) in papers [16, 27, 28, 32, 37, 38, 40, 45],
- Linear discriminant analysis (LDA) in papers [23, 27, 32]
- Quadratic discriminant analysis (QDA) in paper [23],
- Support vector machine (SVM) in papers [6, 9, 16, 18, 21, 24, 27, 28, 36, 38, 41, 42, 44].

**Overview of the papers with DNN used in the classification task**:

- DNN [7, 13, 15, 25].

In the case of deep features, using DNN is the classification already integrated in the DNN network.

### Results

The authors of the analyzed articles used the following *metrics for the evaluation*:

*In the context of image segmentation, the DICE coefficient (score)* has been used to evaluate the similarity between a predicted segmentation mask and the ground truth segmentation mask. Hence, DICE coefficient can be defined [12] as the overlap area of predicted and ground-truth masks divided by the total number of pixels in both images:

1 $$\begin{aligned} DICE= \frac{2 (P \cap GT)}{\left| P\right| +\left| GT \right| } \end{aligned}$$

where GT is the ground-truth mask and P is predicted mask

*To evaluate the classification, the metrics accuracy or/and AUC* have been used. Accuracy is expressed as a proportion of correctly classified subjects among all subjects.

2 $$\begin{aligned} Accuracy={\frac{correct\ classifications}{all\ classifications}} \end{aligned}$$

**Fig. 6:**
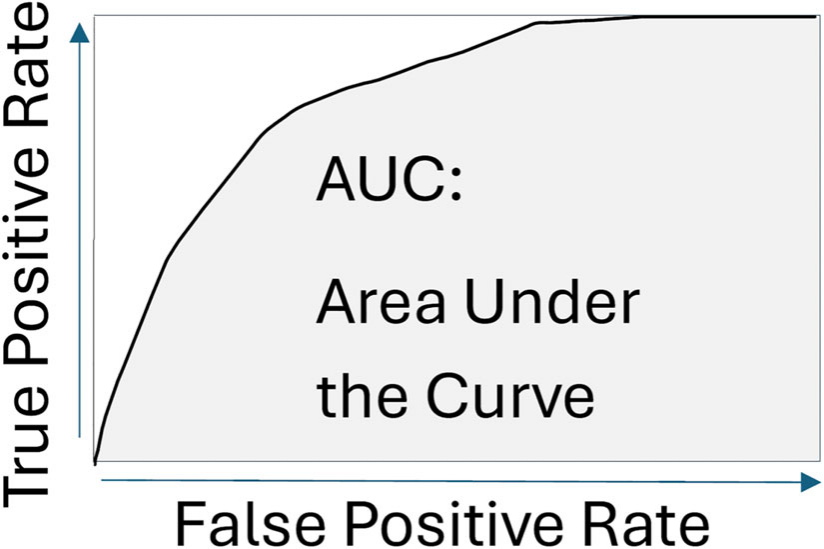
Area under the curve (AUC)

Area under the curve (AUC) is a global measure of diagnostic accuracy that expresses the area under the receiver operating characteristic (ROC) curve (Fig. 6).

AUC helps us estimate how high is the discriminative power of a test [31].

*The overall results achieved in the analyzed papers* are high, e.g., accuracy up to 96% [7] and AUC up to 0.99 [2], which establishes optimism for the successful use of radiomic features. However, it will still be necessary to evaluate extensive datasets so that we can exclude the problem of bias or overfitting.

The results presented in Table 2 show the high accuracy achieved in tasks with PA radiomics. In the table, we merged research tasks with similar objectives and selected the most successful result, which is shown in the table.

However, it should be noted that these results need to be interpreted in relation to the size of the dataset as well as the complexity of the task itself. The comparison is therefore only indicative so that the reader can imagine the current state of knowledge. However, it is not a direct comparison, since the tasks solved could have differed slightly within one group, and especially some datasets may not have been sufficiently representative, which is difficult to assess.

## Discussion

In this article, we analyzed 34 published works that researched the calculation of radiomics using computer vision and machine learning methods in tasks related to PA. For our review, we thoroughly searched for articles on Google Scholar and the PubMed server. When we searched the keywords “(pituitary adenoma) AND (radiomic)” on PubMed, we found only publications in the time frame 2018–2024, and therefore, the focus of our state-of-the-art analysis is based mainly on publications from 2018 to 2024. These publications have been supplemented with papers from 2010, 2011, and 2012 to illustrate the preliminary onset of the use of PA radiomics. However, in these three papers, only a simple semi-automatic segmentation and only one radiomic feature were used: the volume of the adenoma.

The initial increase in advanced scientific studies on PA using radiomics since 2018 is then related to the creation of the PyRadiomics library, which made it easy to use radiomics in software implementations of experiments and was widely used.

**Advantages and disadvantages of the analyzed methods**

The presented methods show very promising results in the whole broad spectrum of researched tasks and objectives related to PA. This is captured in Table 2. As we presented in Fig. 3, there are three basic approaches, each with its advantages and disadvantages.

- **Semantic manually acquired radiomic features** As advantage of the manually acquired features could be seen in the fully supervised approach by radiologists. However, they outweigh the disadvantages and they are the highly time-consuming manual work of the doctor, very low support from computer processing, and only simple processing methods used. In the analyzed works, manual or semi-automatic segmentation was used in most of the works: in 27 studies out of a total of 34.
- **Handcrafted radiomic features algorithm** The advantage of the algorithmic calculation of radiomic features is the support of computer calculations. Compared to the manual approach, it is much more efficient. When compared to deep learning methods, they make do with a smaller amount of data for training the classifier. Another advantage appears to be their interpretability in the case of a smaller number of selected radiomic features. The disadvantage is the necessity of the correct design of which features to calculate with which algorithm. This requires experienced experts in their design. That is why we call them handcrafted features. The expert’s experience through machine learning is also the subsequent choice of a suitable classifier. In the vast majority of analyzed studies, 26 out of 34, the algorithmic calculation of handcrafted features was used. Typically, the PyRadiomics library [34] was used for the calculation. PyRadiomics is written in Python and contains many radiomics calculation algorithms.
- **Deep radiomic features using deep learning** A significant advantage of this approach is that it can greatly overcome the accuracy of results in both segmentation and classification tasks compared to previous methods. Another advantage is that there is no need to design feature calculation algorithms, as the deep neural network derives them on its own. The disadvantage is the need for large datasets for training; the main disadvantage is their poor interpretability. Deep radiomics methods have appeared in studies in recent years, specifically in 4 out of 34 analyzed articles. Notably, among the best results in Table 2, deep radiomics appears 3 times.

**Challenges of deep radiomic features using deep learning (DL)** Regarding prospective DL methods, several challenges are considerable to mention:

- It is important to create high-quality and sufficiently extensive *datasets* necessary for training deep neural networks. Currently, there is no publicly available dataset on radiomics in PA. These datasets can also be augmented using synthetic data generated by generative neural networks.
- *Interpretability* of deep radiomics is a big open challenge. It is necessary to develop and verify methods that will explain to us how deep radiomic features reflect various physics-explainable aspects. Interpretability is also important for checking whether the so-called “Clever Hans effect” [35].

Based on the analyzed works, we summarize that research in the field of radiomics of PA is widely developed, and over time, we can expect a shift to clinical practice for the support of diagnostics. Further research can be envisaged in the wider application of deep learning, including interpretability.

## Data Availability

In the reviewed articles, there is a description of the data used and how they are accessible. This survey study did not work with additional data.
